# The causes of falls: views of older people with visual impairment

**DOI:** 10.1111/hex.12355

**Published:** 2015-03-04

**Authors:** Caroline Brundle, Heather A. Waterman, Claire Ballinger, Nicola Olleveant, Dawn A. Skelton, Penelope Stanford, Chris Todd

**Affiliations:** ^1^ University of Manchester Manchester UK; ^2^ NIHR CLAHRC Wessex Faculty of Health Sciences University of Southampton Southampton UK; ^3^ Glasgow Caledonian University Glasgow UK

**Keywords:** causes of falls, focus groups, interviews, qualitative research, risk factors of falls, severe sight impairment

## Abstract

**Background:**

Sight impairment increases with age and, compared with the general older population, older people with sight impairment are more likely to fall. There is a growing body of evidence on the views and perceptions of older people about falls, but little is published on the views of older people with sight impairment.

**Objective:**

To explore what older people with sight impairment believe to be the causes of falls.

**Design:**

A qualitative design was used, incorporating focus groups and interviews in which participants discussed falls and falls prevention. Framework analysis was employed to identify themes arising from participants' discussions of the causes of falls.

**Setting and participants:**

Fifty‐four community dwelling men and women with sight impairment, aged 65 and over, were recruited from across Greater Manchester, UK.

**Results:**

Five types of factors were identified that were believed to cause falls: (i) health issues and changes in balance caused by ageing; (ii) cognitive and behavioural factors; (iii) the impact of sight impairment on getting around the home; (iv) the impact of sight impairment on negotiating the environment away from home; and (v) unexplained falls.

**Discussion and conclusions:**

Older people with sight impairment reported many researched risk factors previously identified by older people without sight impairment but also described many perceived risks unique to people with sight impairment. There are few interventions to prevent falls aimed at older people with sight impairment, and the results of this study allow further tailoring of such interventions based on views of older people with sight impairment.

## Background

In 2010, it was estimated globally that 39 million people were blind with a further 285 million living with visual impairment.^1^ In the UK, one in eight people aged over 75 and one in three aged over 90 live with sight impairment (binocular visual acuity worse than <6/18 Snellen, >0.5logMAR).^2^, ^1^, ^2^ Preventing falls and promoting safe locomotion presents a particular challenge for older people with poor sight. Evidence suggests sight impairment in older people is associated with increases in the incidence of falls and hip fractures.^3^ Compared to the general older population, this group is 1.7 times more likely to fall, 1.9 times more likely to have multiple falls and 1.3–1.9 times more likely to experience hip fractures.^4^

A recent study also found that older people with eye diseases (age‐related macular degeneration, glaucoma, and Fuchs corneal dystrophy) were around three times more likely than those with good vision to limit their activities due to a fear of falling.^5^ However, this cautiousness may actually increase their risk of falling, as other research has found that people with sight loss who did not take part in physical activity were three times more likely to fall.^6^

Poor visual contrast sensitivity and acuity, reduced visual field and decreased depth perception as well as environmental factors, including poor lighting, are known to be associated with an increased risk of falls amongst older people.^7^, ^8^ The effect of different types of eye conditions upon falling is likely to be important but is not well understood.^7^

Falls often are caused by more than one risk factor,^9^ and this is particularly the case for older people with poor vision. Sight loss can influence a person's balance, movement and the strategies used to negotiate the environment. People with poor vision can lack stability when stepping up and down and adopt different gaits when avoiding obstacles, thus increasing the risk of trips and falls.^10^, ^11^, ^12^

European research has found people deny their own risk of falling and see it as a problem for other people, regardless of their age.^13^, ^14^ Although, there is much research on the causes and impact of falls from many countries, less work has been done to investigate the views of older people with visual impairment on falling.

Thomas Pocklington Trust^15^ (a UK charity that supports people with sight loss) calls for research into the links between sight loss and falls, including the need for the views and experiences of people with sight impairment. This report aims to address the gap in knowledge by investigating what older people with sight impairment consider are the causes of falls.

This research was undertaken within the context of a wider mixed methods project designed to investigate how best to promote adherence to falls prevention interventions. The first phase was qualitative research with one group of older people with sight impairment. The second was a feasibility randomized controlled trial (RCT) with a separate group of participants, which included qualitative interviews on completion of the trial. This report draws on both qualitative elements of the study. The RCT is reported elsewhere.^16^

## Method

### Design

Qualitative research methods illuminate how phenomena are understood, take account of context, and produce explanations which are rich and detailed.^17^ Semi‐structured interviews and focus groups were used in this study to explore experiences, views and concerns, to clarify why particular views were held, and also to facilitate discussion amongst focus group participants.^18^ Key questions were asked in the same way each time, with probing for further information,^19^ giving enough focus to the data collection while still allowing participants opportunities to discuss issues of particular personal concern.

In Phase 1, focus groups took place with older people with sight impairment and their carers. Those not able to participate in groups were invited to contribute their perspectives through semi‐structured interviews. Every effort was made to encourage older people with sight impairment to attend focus groups, including offering practical support such as booking transport. However, for some potential participants, their vision, health conditions and other difficulties made them reluctant to attend. By offering interviews in the home as an alternative, we were able to capture the views of a broader range of older people than if focus groups alone were used.

In Phase 2, semi‐structured interviews were used. Focus groups were not practical for this phase because the interviews were conducted as each individual completed their participation in the RCT.

### Participants

Participants were recruited to the study in two ways.^16^ (i) Patients attending Manchester Royal Eye Hospital's low vision clinic were informed about the research by optometrists. (ii) Participants of social and support group meetings of vision‐related charities in Greater Manchester, UK, were invited to participate by one member of the research team (CBr) after giving a presentation about the study.

The RCT eligibility criteria^16^ were applied to recruitment for both phases of the study: moderately or severely vision impaired, defined as visual acuity less than 0.6 logMAR and/or moderate visual field loss, defined as affecting more than 20% of the test location used in a binocular Esterman test;^3^ aged 75 and over (Phase 1), subsequently reduced to 65 and over for Phase 2 due to recruitment difficulties; independently community dwelling; able to walk around their own residence; cognitively able to participate in the study; and able to understand the study requirements. Older people were excluded if they were receiving a home safety or exercise intervention or were cognitively impaired.

People meeting the eligibility criteria who were interested in taking part in the research were given a participant information sheet (PIS) to take away and consider. The PIS was appropriately designed for people with sight impairment, with columns of large print on a yellow background. A follow‐up telephone call was made a few days later to answer questions and confirm participation.

Phase 1 took place between September and December 2011. Four focus groups were carried out. Group size varied between three and eight participants to facilitate discussion and turn taking, which can be difficult when visual conversation cues cannot be relied upon. Six people participated in semi‐structured interviews, three with a carer present.

Thirty‐three people participated in semi‐structured interviews in Phase 2, two with a carer. These interviews took place between October 2012 and May 2013.

### Data collection

All participants provided written informed consent. Focus groups took place at a clinical research facility which was accessible for those with impaired mobility. The groups were facilitated by experienced qualitative researchers (CB, PS, HW). Four other members of the research team were present in order to meet participants as they arrived, to provide assistance in navigating around the room, help with turn taking during the discussions and arrange transport home.

We drew on expertise from within the project management team, took advice from the Thomas Pocklington Trust and iteratively built on our early experiences of the groups to develop effective strategies for eliciting and promoting discussion. For example, wearing of brightly coloured clothes by researchers, each in a different colour, to help participants to distinguish between them, and making available a hearing loop system for hearing aid users.

Semi‐structured interviews were carried out by experienced individual researchers (CB and NO). The topic guides used were developed by the project management team. Questions about the causes of falls were asked as part of wider discussions around sight impairment and falls prevention.

### Data analysis

Focus group and interview data were audio‐recorded, transcribed and anonymized. Framework analysis was used, selected for its explicit and systematic process of sorting and ordering data, thus making the resultant findings accessible. The framework approach comprises five interconnected phases:^20^

1. Familiarization with the data to gain an overview of the data coverage.
2. Identifying a thematic framework, drawing upon recurrent themes and issues introduced through the topic guide. Themes are grouped under a smaller number of higher order main themes.
3. Indexing data into the framework, deciding which themes apply to each phrase or paragraph in the data.
4. Developing charts from categories identified in the framework so that each main theme and its subtopics are plotted in separate charts.
5. Mapping and interpretation, in which key points of each piece of data are summarized into the thematic matrix, retaining the context and language in which it was expressed.

The use of the NVivo 9 software package facilitated the analysis.

Analysis of the two phases of qualitative research produced similar themes so the findings are presented in the results section without differentiation.

## Results

Fifty‐four older people with sight impairment took part in the study, 38 women and 16 men. The mean age was 83 (range 65–96). The characteristics of the research participants are shown in Table 1.

**Table 1:** Characteristics of focus group and interview participants

Method	Number of OPVIs	Number of NoKs	Gender	Ethnicity	Mean age (years)
Phase 1 FG1	2	1	OPVI: M = 1, F = 1 NoK: M = 1	WB	81
Phase 1 FG2	6	2	OPVI: M = 1, F = 5 NoKs: M = 2	WB	85
Phase 1 FG3	4	2	OPVI: M = 1, F = 3 NoKs: M = 1, F = 1	WB	84
Phase 1 FG4	3	1	OPVI: F = 3 NoK: M = 1	OPVI: WB = 2, White Irish = 1 NoK: WB = 1	81
Phase 1 Ints OPVIs	3	N/A	F = 3	WB	83
Phase 1 Ints OPVIs + NoKs	3	3	OPVI: M = 1, F = 2 NoKs: M = 2, F = 1	OPVI: WB = 2, White Other = 1 NoKs: WB = 3	87
Phase 2 Ints OPVIs	31	N/A	M = 10, F = 21	WB = 29, White Irish = 1, White Other = 1	82
Phase 2 Ints OPVIs + NoKs	2	2	OPVI: M = 2 NoKs: F = 2	WB	83
Total	54	11	OPVI: M = 16, F = 38 NoKs: M = 7, F = 4	OPVI: WB = 50, White Irish = 2, White Other = 2 NoKs: WB = 11	83

FG, focus group; Ints, interviews; OPVI, older people with visual impairment; NoK, next of kin or carer; M, male; F, female; WB, White British; N/A, not applicable.

Five themes capturing older people's views on the causes of falls were identified in the analysis and are described in this section.

1. Health issues and changes in balance caused by ageing.
2. Cognitive and behavioural factors, including risk taking.
3. The impact of sight impairment on getting around the home.
4. The impact of sight impairment on negotiating the environment away from home.
5. Unexplained falls.

The descriptions of the themes are elaborated with extracts from the interview and focus group transcripts (annotated as Int and FG respectively below).

### Health issues and changes in balance caused by ageing

Older people with sight impairment suggested several health issues that they believed were responsible for causing them and other older people to have falls. Some of the suggestions were specific conditions and related symptoms such as Parkinson's Disease, arthritis, vertigo and a lack of sensation in the feet caused by diabetes. Older people also described more vague health‐related factors they linked with falling, such as knees ‘giving out’, poor circulation, heart problems, blacking out and ear problems that cause dizziness. Some suggested that medication taken to control health problems, rather than the health conditions themselves, could be responsible for causing people to fall.

Sometimes my knee, this knee like, you know, on my right leg like, that's a little bit dodgy now and again, and sometimes a factor. (Phase 2 Int P001)

When you sit down or lie down your circulation goes a little bit*…*you must make sure you shake yourself around to get that circulation going. (Phase 1 FG1)

Balance problems caused difficulties for many participants, and some of them talked about other older people being more likely to fall as a result of being ‘wobbly’ and unsteady on their feet. Turning around too quickly caused some participants to fall.

Well I fell over, I swung around quickly, which is sometimes the reason why people fall, or they get up quickly from being sat down, and they're dizzy from that. Because I swung round quickly having bent down to pick the washing up, and fell over the basket! (Phase 2 Int P042)

It was suggested that ageing and losing balance were unavoidably linked.

Once you are past a certain age you start losing your balance really; there's nothing you can do about it. (Phase 2 Int P020)

Others believed standing up too quickly or not getting well balanced after standing up prior to walking resulted in falling.

If the phone rings or the doorbell rings, you get up quickly to answer the phone and you go giddy, I know I do. (Phase 2 Int P038)

I found that I'd gone to sleep and the television was still on. I jumped up. It was very bad to do that because I got to about there [pointing to wall]. I'd turned the television off, but I don't remember much after. I remember being near the wall, and sliding down the wall, but I blacked out. (Phase 1 Int P04)

### Cognitive and behavioural factors

Many older people with sight impairment blamed falling on carelessness and rushing unnecessarily when out and about or at home, for example to get to the telephone or door. This was sometimes talked about in the context of vision loss, but often was just discussed in relation to ageing.

You think you can do better than what you can*…*I think a lot of people do that, you know, you think you're still 20. Because your mind doesn't…well, it does alter because you forget things, but it doesn't really accept that you are as old as you are, you know… (Phase 1 Int P06)

Rushing and not taking care were frequently given as the reasons why ‘they’ – other older people rather than themselves – would fall.

They don't slow down; they try to carry on the same pace as they used to do. (Phase 2 Int P027)

I think it's just carelessness really. (Phase 2 Int P035)

Several participants believed that using some general common sense, taking care and slowing down, they were less likely to fall than those others who rushed around. However, risk‐taking behaviour was practiced by others, such as climbing onto chairs to reach items on high shelves, even though they acknowledged they knew they really should not be doing such things.

You've just got to think what you're doing all the time, haven't you? (Phase 1 FG 4)

Sometimes I climb up and I shouldn't. I know that. That's my fault. (Phase 1 Int P05)

I actually fell over in the garden, missed my step, and broke my wrist*…*I was stood on a wall and… doing… cutting some nettles down or ivy, that's right, and I stepped back and missed my step and woof, off I went, and I went back like that. (Phase 2 Int P09)

Both lack of exercise and exercising were implicated in causing falls. A small number of participants recognized that exercising less in older age as could lead to frailty and falling.

*…*exercise and going out and about and what not, if you don't do that then*…*they're more likely to fall. (Phase 2 Int P034)

Some older people with sight impairment were concerned that exercise programmes designed to reduce the risk of falls could actually be counterproductive.

You might fall while you're doing your exercises. (Phase 2 Int P029)

### The impact of sight impairment on getting around the home

Many older people with sight impairment talked about falls in the home as a result of tripping on unseen hazards.

It was in the bedroom and you know the open end of a duvet, I got my foot caught in the end and I went flying and I hurt my shoulder and I had physio for about six months on that shoulder. (Phase 2 Int P034)

Well, of course, rugs in the house, tripping over rugs and things like that, or in the house, the carpet, when you've got a divider, going from one room to the other, if they come a bit loose and you stub your toe against them, things like that. (Phase 1 FG2)

Slipping on spillages, recently mopped floors and washing machine leaks that the older people were unable to see had caused falls in the home.

If you spill anything, which I do, because I'm always independent, I can do it [clear up the spillage]. And of course you can't always do it, so you spill something then you don't know where you've spilt it, you don't know where it's landed. And by the time you've found it you've slipped in it. (Phase 1 FG2)

Stairs were seen as a particular problem. Many participants recalled times that they had stumbled or fallen when they thought they had reached the top or bottom of a flight of stairs, but in fact they still had one step to go. Poor lighting was thought to be a contributory factor in some of these cases. There was not agreement on whether going up or going down stairs was more risky for older people with sight impairment; both directions were mentioned as hazardous.

Going down the stairs and it's the last step…the stair carpet was the same colour as the hall carpet, you see, so it all just merged in, and it disappeared completely. It was a horrible feeling. (Phase 1 FG 3)

But it's notable particularly when I'm going upstairs, and I have to be very careful that I don't fall backwards. (Phase 2 Int P039)

Changes to a previously familiar home environment were thought to be particularly hazardous. For example, if visitors moved furniture or left objects on the floor, an older person with sight impairment would be very likely to trip and fall over them because they would not expect them to be there and often navigate around their own home by relying on familiarity rather than sight.

Another reason why we fall is objects in the way, things in the middle of the room, if children have been and left toys. (Phase 2 Int P038)

Some participants reported falling after their home had been altered with the aim of improving safety and reducing the risk of falls. For example, a rug was removed for safety because it was a trip hazard; however, the older person used it as a way of knowing her position in the room and after it was removed fell getting to her bed.

I was amazed on a night how I couldn't find the… It's like it's a bed, it's big enough to see, isn't it? And I couldn't see it, because I'd shifted the mats (Phase 2 Int P005)

### Impact of sight impairment on negotiating the environment away from home

Some older people with sight impairment avoided going out on their own because they were scared about falling while away from their home. Many hazards in the outdoors environment were identified. Of great concern was the state of the pavements. Older people with sight impairment wished that the council would maintain them better to prevent themselves and others from falling while outside. Broken paving slabs, pavements made uneven by tree roots and unfinished maintenance work all made pavements serious trip hazards.

…the pavements are so bad*…*And I just looked up, and whilst I was looking up I went flying and it was over this*…* actually what it was I was looking at a piece of piping sticking out of the*…* you know, not the pavement. It was actually some land that I was walking on, they had taken something away and put soil with gravel over it, and I had to walk across it to get to the pavement on the other side. (Phase 2 Int P016)

I don't know about all pavements, but round here, a lot of the pavements are very uneven*…*I think it's, you know, that where the pavements are breaking up, and sometimes I'm really afraid of it. (Phase 2 Int P002)

In addition to the conditions underfoot, dustbins left out, shops' signs and products, and hedges that have not been trimmed back are all extra obstacles for an older person to deal with when walking on pavements. Some participants were concerned that and if they cannot be seen properly, collisions with these items would cause them to fall.

Some of the shops insist they put all their wares outside all over the pavements*…* oh they're a pain*…*and it is coming further out and further out. (Phase 1 FG2)

I never saw, well, I should have been looking, but I never saw the thing, and I tripped over one of them signs on the pavement. But there was two people that came along and lifted me up. (Phase 2 Int P001)

Moss, gravel, wet leaves and litter had all caused participants to slip and sometimes fall outdoors.

One of the worst ones is carrier bags, on the floor, slippy bags. (Phase 2 Int P038)

In much the same way that stairs indoors can cause problems for older people with sight impairment, many participants identified steps outdoors as a falls risk. They also reported tripping over kerbs when crossing the road and problems caused by steps getting on and off buses.

I definitely fell off the step outside*…* I didn't know it was there. And all of a sudden you've just gone, you know? (Phase 2 Int P033)

There was a railing set in like a kerb, and sort of at the end when the railing went down there was about another six inch of kerb still sticking out. Well as I walked round, I thought the end of the rail was the end of the block, so as I walked forward I tripped over this six inch bit of kerb. (Phase 1 Int P02)

Ramps are often put in place to help people with disabilities; however, some older people with sight impairment found they actually could be more of a hazard than steps. Slopes can be harder to differentiate, for people with poor vision, than steps. Changes to well‐known places have resulted in accidents when familiarity has caused an older person to be less vigilant.

My biggest advantage is knowledge of a place, cognitive mapping, that's my biggest advantage. But it can also mean that you lead to making mistakes because you're anticipating something and it's changed. (Phase 2 Int P022)

### Unexplained falls

A couple of participants were at a loss to explain why they had fallen.

I don't know why I keep falling. Half the time I don't remember falling. I mean when all these falls started, I knew when I was going to go and couldn't stop myself. But now it seems as if I just go, as if I blackout or something like that. That's what's worrying me now*…* (Phase 2 Int P004)

Some older people accepted falling as an inevitable consequence of ageing.

I don't think you can predict it. (Phase 2 Int P017)

I don't think there's anything you could do. (Phase 2 Int P049)

## Discussion

This study has generated new knowledge in a previously unexplored area on the causes of falls from a group of community dwelling older people with sight impairment about two‐thirds of who reported having had at least one fall. Participants confirmed many of the risk factors identified by quantitative prospective studies as having strongest association with falling including sight impairment, gait problems, medical conditions and vertigo.^21^ Many of these risks were incorrectly seen, by them, as inevitable aspects of ageing.

Participants also discussed the impact of sight impairment and hazards in the environment which led to falls including loose carpets or mats, unfamiliar objects on the floor, slippery surfaces, and stairs/steps inside and outside the home, also previously found in research.^8^ The evidence on falls suggests that a hazardous environment is not a risk factor for falls per se; that is if a person's home or outside environment is unsafe, it does not necessarily mean they will fall.^8^ What appears to be significant is the person's ability to cope with an unsafe environment, that is the interaction between the person themselves and the environment.^8^ Thus, older people with sight impairment may be less likely to be able to manage an unsafe environment as they may be unaware of hazards until it is too late. For example, as some of the respondents reported, they found it difficult to locate or were unaware of spillages and fell because of them. If the person does not interact with the environment much because they are not very mobile, the fact that the environment is hazardous is not a huge problem as they would not be placing themselves in danger often. In contrast, a mobile individual with sight impairment who interacts with the environment a great deal is more likely to place themselves in a hazardous situation which could lead to a fall.^8^ Participants indicate that falls were caused by unfamiliar changing environments especially outside the home. The findings add weight to the hypothesis that falls prevention interventions that promote a familiar and safe environment may be successful in preventing falls in those with sight impairment.

The strength of asking patients with sight impairment for their views on the causes of falls is that it provides personal explanations for falls and the context to falling. The meta‐narrative (or big story) in the interviews was that there is a varying, complex, interplay of many factors which may determine whether someone falls and whether they are amenable to preventative interventions. The qualitative data add the ‘human nature’ or behavioural aspect of falling which the epidemiological studies on risk factors for falls cannot. By finding out from patients their experiences of falling, health‐care professionals understand in what circumstances patients fall and whether they take risks, tend to be careless and/or rush and this information becomes a point of reference in planning and evaluating personalized falls prevention interventions. However, tools for assessing the safety of people's homes tend to focus on the material aspects of the person's home, for example whether there any physical trip hazards and do not explicitly interrogate the human context of promoting safety.^22^ Further research is required to develop and test these tools to take into account in what circumstances people fall including behavioural aspects related to falling.

Participants often describe the causes of falls as inevitable and largely beyond their control, for example from blackouts or uneven pavements. Of note, was the omission of lack of exercise as a contributory factor in falls despite some of the sample having participated in an exercise intervention in the RCT to prevent falls. This is further evidence that reinforces the view that many patients think they cannot do much about falling. Espoused beliefs about falls need to be checked against the circumstances of falls and evidence as there might be possible interventions to prevent falls in the future. For example, help with medication adherence may reduce the likelihood of falls from poorly controlled medical conditions. If lack of exercise is not seen as a cause of falls, then this may contribute to poor adherence with exercise programmes.^23^ Further research is required to learn whether exercise programmes have any effect in preventing falls in older people with sight impairment.

Alternately, there were circumstances in which patients in our study report frankly that they were responsible for falling from, for example rushing to answer the door or climbing walls to prune hedges. Hurrying has been identified previously as a risk factor for falls in those with multiple sclerosis^24^ and also in the context of pedestrian road crossings.^8^ Thus, a detailed health‐care assessment of the circumstances of the fall is necessary to understand whether an individual accepts they may fall and/or whether they lack help. Our findings confirm those of another study in which participants who had sustained a fractured hip from falling and who described how they had no choice but to carry out essential household jobs as they had no one to help even though they knew they may be placing themselves in danger of a fall.^25^ This information could help the health‐care professional to discuss with the older person with sight impairment whether there are any ways in which they could make themselves safer, for example by installing a visitor microphone/speaker system at the door so they can let the caller know it will take a while to get there. Other research suggests that this needs to be carried out in an empathetic manner such that it is not patronizing nor feels distressing to the person concerned.^26^, ^27^, ^28^ Further research is required to investigate from the perspective of older people with sight impairment their own personal understandings of how they prevent falls.

## Limitations

The sample were drawn from urban areas and did not represent the experiences of people living in rural areas who might have different experiences outside of the home, for example with lack of public transport and lack of pavements. We included people who had not fallen and participants had received either (i) a home safety assessment and modification or (ii) home safety assessment and modification, and home‐based exercise programme as part of the RCT. These could be construed as limitations of the study, but it means the sample is inclusive of a broad group of older people with sight impairment making the findings relevant to a bigger section of the older population with sight impairment. Another limitation of our sample was the absence of participants from ethnic minorities. They were not actively excluded, but it transpired that they did not attend low vision clinics nor were members of the vision‐related charities, and hence, they were not recruited. Novel methods to access this group of older people with sight impairment are needed in future research.

## Conclusion

Older people with sight impairment identified many factors which have previously been reported as risk factors for falls. They particularly emphasized environmental hazards, in causing falls and stressed that sometimes they or others took unnecessary risks, rushed or were careless which also led to falls. This confirms the general view that it is not a hazardous environment per se which causes falls but how a person interacts with it. Furthermore, the findings from the study suggest this group was relatively mobile around the inside and outside of their home and were potentially placing themselves in danger. Very few identified lack of exercise as a causative factor and rather suggested it could cause more falls as it would make people more mobile. There does seem to be evidence which supports findings from other research to suggest that assessment and modification of the environment would be a useful intervention to prevent falls in this group^23^ but, specifically, our study found there also needs to be a behavioural assessment including risk taking and freedom of individuals in terms of preventing falls. Although health‐care professionals may already assess espoused beliefs and behaviour surrounding the circumstances of falls, these factors do not feature in existing home safety assessment tools for older people with sight impairment. Further research is required to ascertain how this behavioural assessment may be incorporated into such tools. More research is required to investigate whether falls prevention programmes for older people with sight impairment should focus on home safety assessment and modification and/or exercise programmes.

## Sources of funding

This article represents independent research commissioned by the National Institute for Health Research (NIHR) under the Research for Patient Benefit Programme (RfPB), reference number: PB‐PG‐0909‐20090. The views expressed in this publication are those of the author(s) and not necessarily those of the NHS, the NIHR or the Department of Health. The research was also financially supported by the Thomas Pocklington Trust.

## Conflict of interests

No conflict of interests have been declared.

## Notes

1 Snellen test: an eyesight test using rows of letters printed in successively decreasing sizes (the Snellen scale) of which patients are asked to read as many as they can.

2 LogMar: an eyesight test in which the letters decrease geometrically.

3 Esterman binocular test: an eyesight test on both eyes together where the person wears their usual distance glasses.

## References

[1] World Health Organisation (2010) Global data on visual impairments. Available at: http://www.who.int/blindness/GLOBALDATAFINALforweb.pdf?ua=1, accessed 20 December 2014.

[2] Evans JR , Fletcher AE , Wormald RPL *et al* Prevalence of visual impairment in people aged 75 years and older in Britain: results from the MRC trial of assessment and management of older people in the community. British Journal of Ophthalmology, 2002; 86: 795–800. 12084753 10.1136/bjo.86.7.795 PMC1771210

[3] Crews JE , Campbell VA . Vision impairment and hearing loss among community‐dwelling older Americans: implications for health and functioning. American Journal of Public Health, 2004; 94: 823–829. 15117707 10.2105/ajph.94.5.823 PMC1448344

[4] Legood R , Scuffham P , Cryer C . Are we blind to injuries in the visually impaired? A review of the literature. Injury Prevention, 2002; 8: 155–160. 12120837 10.1136/ip.8.2.155 PMC1730864

[5] Dhital A , Pey T , Stanford MR . Visual loss and falls: a review. Eye, 2010; 24: 1437–1446. 20448666 10.1038/eye.2010.60

[6] Lord S , Sherrington C , Menz H , Close J . Falls in Older People: Risk Factors and Strategies for Prevention, 2nd edn Cambridge: Cambridge University Press, 2007.

[7] Rubenstein LZ . Falls in older people: epidemiology, risk factors and strategies for prevention. Age and Ageing, 2006; 35 (Suppl 2): ii37–ii41. 16926202 10.1093/ageing/afl084

[8] Buckley JG , Heasley KJ , Twigg P , Elliott DB . The effects of blurred vision on the mechanics of landing during stepping down by the elderly. Gait & Posture, 2005; 21: 65–71. 15536035 10.1016/j.gaitpost.2003.12.001

[9] Buckley JG , Heasley K , Scally A , Elliott DB . The effects of blurring vision on medio‐lateral balance during stepping up or down to a new level in the elderly. Gait & Posture, 2005; 22: 146–153. 16139750 10.1016/j.gaitpost.2004.08.006

[10] Buckley JG , Panesar GK , MacLellan MJ , Pacey IE , Barrett BT . Changes to control of adaptive gait in individuals with long‐standing reduced stereoacuity. Investigative Ophthalmology & Visual Science, 2010; 51: 2487–2495. 20335609 10.1167/iovs.09-3858

[11] Wang MY , Rousseau J , Boisjoly H *et al* Activity limitation due to a fear of falling in older adults with eye disease. Investigative Ophthalmology & Visual Science, 2012; 53: 7967–7972. 23132799 10.1167/iovs.12-10701

[12] Lamoureux E , Gadgil S , Pesudovs K *et al* The relationship between visual function, duration and main causes of vision loss and falls in older people with low vision. Graefes Archive for Clinical and Experimental Ophthalmology, 2010; 248: 527–533. 10.1007/s00417-009-1260-x 20054556

[13] Yardley L , Donovan‐Hall M , Francis K , Todd C . Older people's views of advice about falls prevention: a qualitative study. Health Education Research, 2006; 21: 508–517. 16467173 10.1093/her/cyh077

[14] Yardley L , Bishop FL , Beyer N *et al* Older people's views of falls‐prevention interventions in six European countries. The Gerontologist, 2006; 46: 650–660. 17050756 10.1093/geront/46.5.650

[15] Martin M . Falls in Older People With Sight Loss: A Review of Emerging Research and key Action Points. London: Thomas Pocklington Trust, 2013.

[16] Waterman H , Ballinger C , Brundle C *et al* Adapting a home safety programme and an exercise programme to prevent falls in a feasibility study. Presentation at Royal National Institute for the Blind, London, 21st October, 2014.

[17] Mason J . Qualitative Researching, 2nd edn London: Sage Publications, 2002.

[18] Morgan DL . Focus Groups as Qualitative Research, 2nd edn Thousand Oaks, CA: Sage Publications, 1997.

[19] Arthur S , Nazroo J . Designing fieldwork strategies and materials In: Ritchie J, Lewis J (eds) Qualitative Research Practice. London: Sage Publications, 2005: 109–137.

[20] Ritchie J , Spencer L , O'Connor W . Carrying out qualitative analysis In: Ritchie J, Lewis J (eds) Qualitative Research Practice. London: Sage Publications, 2005: 219–262.

[21] Deandrea S , Lucenteforte E , Bravi F , Foschi R , La Vecchia C , Negri E . Risk factors for falls in community‐dwelling older people: a systematic review and meta‐analysis. Epidemiology, 2010; 21: 658–668. 20585256 10.1097/EDE.0b013e3181e89905

[22] Clemson L . Home Fall Hazards: A Guide to Identifying Fall Hazards in the Homes of Elderly People and an Accompaniment to the Assessment Tool, the Westmead Home Safety Assessment. West Brunswick, Vic.: Coordinates, 1997.

[23] Campbell AJ , Robertson MC , Grow SJL *et al* Randomised controlled trial of prevention of falls in people aged > or =75 with severe visual impairment: the VIP trial. British Medical Journal, 2005; 331: 817. 16183652 10.1136/bmj.38601.447731.55 PMC1246082

[24] Nilsagard Y , Denison E , Gunnarsson LG , Bostrum K . Factors perceived as being related to accidental falls by persons with multiple sclerosis. Disability and Rehabilitation, 2009; 31916: 1301–1310. 10.1080/09638280802532639 19479575

[25] Mahler M , Sarvimaki A . Fear of falling from a daily life perspective; narratives from later life. Scandanavian Journal of Caring Sciences, 2000; 26: 38–44. 10.1111/j.1471-6712.2011.00901.x 21623853

[26] Ballinger C , Payne S . Falling from grace or into expert hands? Alternative accounts about falling in older people. British Journal of Occupational Therapy, 2000; 63: 573–579.

[27] Health Foundation . Available at: http://patientsafety.health.org.uk/sites/default/files/resources/an_overview_of_best_practice_for_falls_prevention_from_an_occupational_therapy_perspective_0.pdf, accessed 20 December 2014.

[28] Age UK . Available at: http://www.ageuk.org.uk/Documents/EN-GB/For-professionals/Research/Services-what_works_spreads.pdf?dtrk=true, accessed 20 December 2014.

